# Occupancy Modeling for Improved Accuracy and Understanding of Pathogen Prevalence and Dynamics

**DOI:** 10.1371/journal.pone.0116605

**Published:** 2015-03-04

**Authors:** Michael E. Colvin, James T. Peterson, Michael L. Kent, Carl B. Schreck

**Affiliations:** 1 Oregon Cooperative Fish and Wildlife Research Unit, Department of Fisheries and Wildlife, Oregon State University, 104 Nash Hall, Corvallis, Oregon, 97331, United States of America; 2 Oregon Cooperative Fish and Wildlife Research Unit, U.S. Geological Survey-Department of Fisheries and Wildlife, Oregon State University, 104 Nash Hall, Corvallis, Oregon, 97331, United States of America; 3 Department of Microbiology, Oregon State University, 220 Nash Hall, Corvallis, Oregon, 97331, United States of America; University of California Irvine, UNITED STATES

## Abstract

Most pathogen detection tests are imperfect, with a sensitivity < 100%, thereby resulting in the potential for a false negative, where a pathogen is present but not detected. False negatives in a sample inflate the number of non-detections, negatively biasing estimates of pathogen prevalence. Histological examination of tissues as a diagnostic test can be advantageous as multiple pathogens can be examined and providing important information on associated pathological changes to the host. However, it is usually less sensitive than molecular or microbiological tests for specific pathogens. Our study objectives were to 1) develop a hierarchical occupancy model to examine pathogen prevalence in spring Chinook salmon *Oncorhynchus tshawytscha* and their distribution among host tissues 2) use the model to estimate pathogen-specific test sensitivities and infection rates, and 3) illustrate the effect of using replicate within host sampling on sample sizes required to detect a pathogen. We examined histological sections of replicate tissue samples from spring Chinook salmon *O. tshawytscha* collected after spawning for common pathogens seen in this population: *Apophallus/*echinostome metacercariae, *Parvicapsula minibicornis, Nanophyetus salmincola/* metacercariae, and *Renibacterium salmoninarum*. A hierarchical occupancy model was developed to estimate pathogen and tissue-specific test sensitivities and unbiased estimation of host- and organ-level infection rates. Model estimated sensitivities and host- and organ-level infections rates varied among pathogens and model estimated infection rate was higher than prevalence unadjusted for test sensitivity, confirming that prevalence unadjusted for test sensitivity was negatively biased. The modeling approach provided an analytical approach for using hierarchically structured pathogen detection data from lower sensitivity diagnostic tests, such as histology, to obtain unbiased pathogen prevalence estimates with associated uncertainties. Accounting for test sensitivity using within host replicate samples also required fewer individual fish to be sampled. This approach is useful for evaluating pathogen or microbe community dynamics when test sensitivity is <100%.

## Introduction

Parasites and pathogens (hereafter collectively referred to as pathogens) are routinely surveyed and monitored in domestic (e.g., fish culture facilities) [1] and free-ranging (hereafter, natural) wildlife populations [2]. Accurate assessment of pathogen presence and prevalence is also relevant for populations destined for geographic translocation and introduction [2–4]. This is especially critical when those introductions may lead to pathogen introductions and potential population declines of endangered species. For example, with salmonid fishes, introduction of the causative agent of whirling disease, *Myxobolus cerebralis*, in the United States intermountain West through introductions of infected fish resulted in population declines and negative biological and economic impacts [5–7]. Similarly, translocations of raccoon *Procyon lotor* captured in Texas and Florida resulted in the introduction of parvoviral enteritis and rabies to recipient populations [8,9]. In an extreme case, the loss of approximately half of Hawaiian avian fauna was attributed, at least in part, to the introductions of avian pathogens [10].

The ability to understand the pathogen-host dynamics and develop effective pathogen mitigation strategies and risk analyses depends upon the quality of the data. Many pathogen detection tests are imperfect (i.e., may not detect a pathogen even if it is present) and therefore pathogen detection probabilities are generally less than one [11]. Imperfect detection increases the likelihood that a pathogen present in a population is missed and induces negative biases in the estimation of pathogen prevalence. Molecular and other similar screening methods that use nonspecific tests (e.g., PCR tests that use universal DNA primers) are particularly vulnerable to false negatives regarding detection of pathogens in low abundance, especially when sympatric with other species that are in much greater abundance, regardless of virulence of any particular pathogen [12–14]. Histopathology has been used in recent surveys for diseases in wild animal populations, particularly for screening of pathogens or non-infectious lesions without *a priori* restrictions [15–20], or when toxicopathic changes were a target endpoint [21,22]. However, a recent evaluation on Chinook salmon *Oncorhynchus tshawytscha* concluded that histological methods were considerably less sensitive than microbiological tests for certain pathogens, resulting in numerous false negatives [17]. The lack of sensitivity of histology compared to microbiological tests that involve either amplification of pathogens in culture or DNA by PCR would certainly apply to other pathogen surveys [13,23]. Imperfect detection of pathogens can substantially influence sample design. Greater sample sizes are required to detect a pathogen in a population, to compensate for false negatives, which may pose a significant constraint if sampling is lethal, something of great importance when lethal take involves threatened and endangered wildlife species. Researchers also have an ethical obligation to minimize lethal sampling of vertebrate species required by Institutional Animal Care and Use Committees [24]. Therefore, analytical approaches that provide accurate estimates of pathogen presence and prevalence while reducing the number of individuals sampled and handled are desirable. Confidence regarding parasite prevalence estimates is also vital for studies concerned with pathogen community ecology.

Test sensitivity is traditionally calculated by comparing the number of detections from one test with those of a high sensitivity “gold standard” diagnostic, such as highly sensitive PCR test, thereby providing an estimate of the probability of detecting the pathogen when truly present [25,26]. The gold standard test is assumed to have perfect pathogen detection (i.e., sensitivity = 100%); however it is possible that these tests are imperfect as well. For example, Kent et al. [17] detected spring Chinook salmon pathogens in histological samples that were not detected in corresponding pathogen-specific gold standard tests recommended by the American Fisheries Society-Fish Health Section [27]. Similarly, false negatives occurred between bacteriological and histopathological tests, the confirmatory tests for bovine tuberculosis [28]. There still remains need for a flexible, cost-effective approach to accurately estimate pathogen prevalence and detection probability (i.e., detecting a pathogen given it is present) that does not require restrictive assumptions. Approaches have been developed to estimate pathogen prevalence in pooled samples using imperfect test [29,30]. Recently occupancy-detection estimation has been proposed as a method to account for imperfect detection of *Plasmodium* species and provide estimates of test sensitivity [31]. Occupancy-detection estimation includes the collection of replicate tissue samples from individual hosts that are used to estimate pathogen detection probabilities and provide better estimates of test sensitivity compared to ad hoc comparisons between diagnostic tests (e.g., a test compared to a gold standard test). Application of occupancy-detection models to the study of pathogens in natural populations has been limited to the individual host-level [32–34]. Additional insight into within host-pathogen dynamics can be gained by evaluating multiple organ tissues within the host in a hierarchical structure. Our study objectives were to 1) develop a hierarchical occupancy model to estimate pathogen prevalence (hereafter infection rate) in spring Chinook salmon and their distribution among host organ tissues 2) use the model to estimate pathogen- and tissue-specific test sensitivities and host- and organ-level infection rates, and 3) illustrate the effect of using replicate within host sampling on number of hosts sampled required to detect a pathogen in a population given it is present (i.e., power). This manuscript reports on the successful achievement of the study objectives.

## Methods

### Ethics statement

This study was performed under the auspices of animal use protocol AUP # 4438. The protocol was approved by the Institutional Animal Care and Use Committee of Oregon State University. This particular study utilized fish that were already dead as a result of routine spring Chinook salmon spawning operations conducted by Oregon Department of Fish and Wildlife. Since this study utilized existing dead fish no field permit was required from state regulatory agencies.

### Fish sampling

Fish were sampled during an ongoing study of adult spring Chinook salmon in the upper Willamette River (UWR), Oregon, in September 2012. The threatened status of UWR spring Chinook under the Endangered Species Act limits lethal sampling of live fish in this system [35]. However, conservation hatchery operations capture spring Chinook at Dexter Dam (Dexter, Oregon; 43.919937N, 122.815739W) and move them to Willamette Hatchery to mature over summer. Oregon Department of Fish and Wildlife (ODFW) hatchery spawning operations allowed us to opportunistically collect a sufficient sample of fresh carcasses and tissues therein for pathogen detection. Estimates of pathogen prevalence from previous monitoring at this location varied from 15 to 95% [17,36] and were used as guides to estimate required sample sizes. An *a priori* simulation evaluation of the sample size requirements indicated that at least 23 fish were needed to obtain a 90% probability of collecting at least one infected fish assuming a parasite infection rate of 10% assuming 100% sensitivity (M. Colvin unpublished simulation data). The number of fish sampled was 26 to ensure adequate sampling and because sufficient numbers of fish were available. Sampled fish were euthanized and spawned by ODFW employees and the carcass necropsied within 30 minutes of death.

### Necropsy, tissue sampling, and histological processing

**Fig 1:**
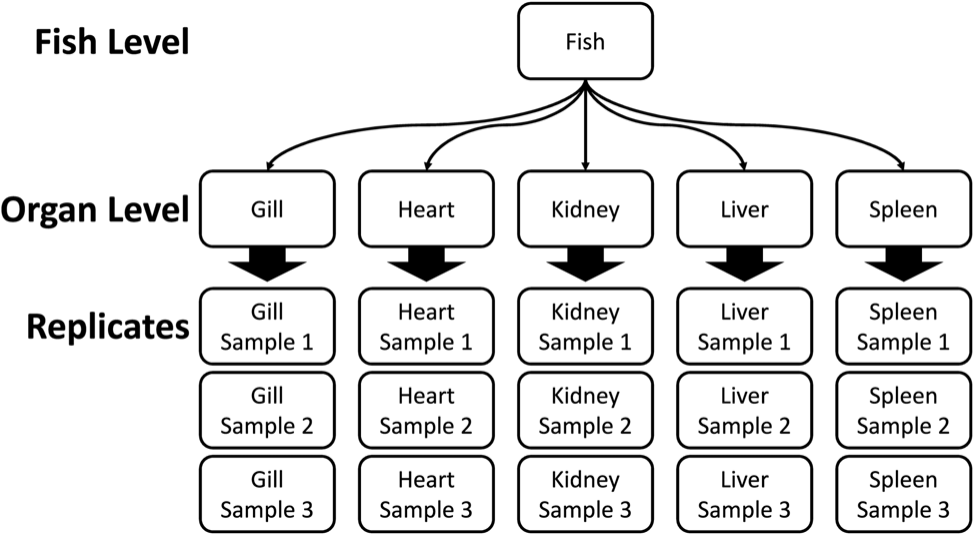
Data structure illustration for pathogens detected by histological analysis of tissues sampled from Willamette Hatchery (Oakridge, OR) spring Chinook salmon. Histological samples were processed to minimize the number of slides needed and therefore multiple tissues were processed on a single slide when feasible.

Tissue samples from organs were collected for histological examination during necropsy. Three tissue samples of gill, heart, posterior kidney, spleen, and liver were removed and immediately fixed in 10% buffered neutral formalin for at least 7 d (Fig. 1). Fixed tissues were trimmed and placed in tissue cassettes, then dehydrated, sectioned, and affixed to slides using standard histological techniques by the Oregon State University Veterinary Diagnostic Laboratory. Processing resulted in 6 slides for each individual fish; 3 slides for visceral organs (heart, kidney, spleen, liver contained on a slide) and 3 slides for gill tissues. Slides were prepared using standard histological protocols and stained with hematoxylin and eosin [37]. All slides were evaluated for pathogen presence by one of the co-authors (M.K.), who is an experienced fish pathologist. Organ-specific pathogen detections were represented as a series of 0’s and 1’s. The following pathogens were encountered in tissue sections in many fish, and thus were included in our analysis: 1) metacercariae in the gill (either *Apophallus* sp. or echinostome type), 2) metacercariae of *Nanophyetus salmnicola* in various organs, 3) *Renibacterium salmoninarum*, diagnosed by the presences of granulomatous lesions with no other associated pathogens in various organs, or 4) *Parvicapsula minibicornis* in kidney glomeruli or tubule tissue.

### Sample and organ unadjusted prevalence

Prevalence unadjusted for test sensitivity, hereafter unadjusted prevalence (α), was calculated for each pathogen encountered. Typical sampling would include a single tissue sample for each organ and those values aggregated to the host-level [17]; however this dataset included 3 organ-level tissue samples (hereafter, replicates). To approximate the typical approach, organ-level and host-level prevalence was calculated for each replicate. Organ-level unadjusted prevalence was calculated for each tissue sample replicate by dividing the number of detections by the sample size for each pathogen detected. For example, if *R*. *salmoninarum* was detected 10, 12, and 9 times (i.e., 3 replicate tissue samples) out of 26 hosts then the prevalence would be 38.4, 46.1, and 34.6% respectively. Host-level unadjusted prevalence was calculated for each replicate by aggregating among organ detections to the host-level and dividing the number of infected hosts by the number of hosts. For example, if a pathogen was detected in kidney and heart tissues these detections were aggregated to 1 at the host- level. These values were then graphically compared to occupancy model estimated prevalence (described below) for each pathogen to examine discrepancies between approaches.

### Pathogen occupancy model overview, fitting, and inference

The hierarchical occupancy model developed in this study estimated 3 quantities: 1) the probability an individual host is infected by a pathogen (infection rate; Ψ); 2) the probability an organ is infected given the host is infected (conditional organ infection rate; φ); and 3) the probability a pathogen is detected in an organ given the organ is infected (sensitivity; *s*) (Table 1). Hierarchical occupancy models incorporate additional information from replicate tissue samples of secondary sampling units, organs within fish in this case (Fig. 1). The model also assumed pathogens were correctly identified when detected and that organ tissue-specific detection probabilities did not depend on overall pathogen burden (i.e., sensitivity was equal for low and high burdens). These assumptions can be relaxed as discussed later. We believe these assumptions were reasonable given the extensive experience of the slide reader, and there was no possibility for morphological changes in organ-specific pathogen occupancy because tissues were preserved in a formalin-based fixative.

**Table 1:** Parameters, symbols, and descriptions of terms used in this study.

Parameter	Occupancy-detection analog	Symbol	Description
Infection rate	Occupancy	Ψ	Probability of a host being infected by a pathogen.
		φ	Probability of an organ being infected by a pathogen given the host is infected.
Prevalence	Proportion occupied units	*P*	Proportion of hosts or organs predicted to be infected calculated as the number of infected divided by the total.
Sensitivity	Detection probability	*s*	Probability of detecting a pathogen in organ tissue samples given the pathogen is present.
Unadjusted prevalence	Naïve prevalence	α	The proportion of hosts in a sample infected by a pathogen calculated as the observed number of infected hosts/number of hosts sampled.

Given the complex nature of the hierarchical modeling framework, we used a Bayesian approach and state space formulation [38] to estimate host and organ-level pathogen infection rates (Ψ and φ) and pathogen- and tissue-specific sensitivities (*s*). Construction of the likelihood of the data given the occupancy model is difficult due to hierarchical dependencies (i.e., organs nested within fish) arising from the sampling process. However, the likelihood of the model given the data can be constructed using hierarchical state space models [38]. These models can be fit using a Bayesian approach to estimate Ψ and φ and *s*.

Host-level pathogen infection state was modeled as a Bernoulli distributed variable that estimated the unobserved infection state for each fish (i.e., uninfected = 0, infected = 1) with probability Ψ_i_ as:

$$Z_{i}\sim Bern({\text{Ψ}}_{i})$$ (1)

where, *Z*_*i*_ was the unobserved infection state for fish *i* and Ψ_i_ is the probability that fish *i* is infected by the pathogen. Infection rate (Ψ_i_) was predicted using a linear model with a logit link as:

$$\text{ln}(\frac{{\text{Ψ}}_{i}}{1-{\text{Ψ}}_{i}})=\omega_{0i},$$ (2)

where, ω_0*i*_ is the intercept of the linear model. Linear models were used in equation 2 and in following equations to facilitate model fitting. The infection rate of organ *j* within fish *i* conditional on fish *i* being infected (i.e., *Z*_*i*_ = 1) was estimated as:

$$z_{ij}\sim Bern(Z_{i}\cdot \varphi_{ij}),$$ (3)

where, *z*_*ij*_ was the unobserved infection state for organ *j* within fish *i*, *Z*_*i*_ was the host-level infection state of the *i*th fish and φ_*ij*_ was the probability that organ *j* was infected given the fish was infected (i.e., *Z*_*i*_ = 1) otherwise φ_*ij*_ = 0. A linear model with a logit link was used to estimate φ_*ij*_ as:

$$\text{ln}(\frac{\varphi_{ij}}{1-\varphi_{ij}})=\beta_{0ij},$$ (4)

where β_0*ij*_ is the intercept of a linear model predicting infection rate for organ *j* within fish *i*.

A detection model was used to link observed tissue level pathogen detections to the process model described above (equations 1–4) as:

$$y_{ijk}\sim Bern(z_{ij}\cdot s_{ij}),$$ (5)

where *y*_*ijk*_ was the observed pathogen detections (0 or 1) for the replicate *k* from organ *j* within fish *i*, *z*_*ij*_ was the unobserved organ-specific infection state for fish *i*, and *s*_*ij*_ was the conditional organ tissue-level sensitivity given the organ was infected (i.e., *z*_*ij*_ = 1), otherwise *s*_*ij*_ = 0. Organ tissue-specific sensitivity (*s*_*ij*_) was predicted using a linear model as:

$$\text{ln}(\frac{s_{ij}}{1-s_{ij}})=\theta_{0ij},$$ (6)

where θ_0*ij*_ was the intercept term for organ *j* within fish *i*

Separate hierarchical occupancy models for each pathogen encountered in the study were fit using Markov Chain Monte Carlo (MCMC) implemented in WinBUGS 2.14 [39]. Each model was fit using diffuse priors and three chains with 130k iterations and 30k burn-in samples (i.e., the first 30k iterations were discarded). Chains were thinned by 10 for *Apophallus/*echinostome metacercariae 20 for *P*. *minibicornis* and *N*. *salmnicola*, and 50 for *R*. *salmoninarum* to minimize autocorrelation in the MCMC. The Gelman-Rubin convergence statistic and visual inspection of the 3 MCMC chains were used to ensure adequate chain mixing was achieved [40]. Posterior predictive check was used to calculate a Bayesian goodness-of-fit (GOF) value for fitted models to assess whether the model adequately fit the data [38,41]. Goodness-of-fit values vary from 0 to 1 with extreme values indicating lack of fit and values close to 0.5 indicating adequate model fit. The R programming environment, WinBUGS 2.14, and the R2WinBUGS package were used for all analyses [39,42,43]. Separate host- and organ-level infection rates could not be estimated for *Apophallus/*echinostome metacercariae because this parasite was only detected in gill tissue. Therefore, the organ-level infection rate was set to one during model fitting to estimate a host-level infection rate and prevalence. Further information regarding the occupancy model and example code and data for replicating the analysis are available in S1 Information.

### Effects of replicates and hierarchical sampling on pathogen detection

The approach developed in this study provides a framework to analyze pathogen detection data from multiple tissue types within a host and replicate tissue samples surveyed, which represents a tradeoff between sampling more primary sampling units (i.e., hosts) or more intensive tissue sampling within hosts (i.e., > 1 replicate). Because the primary objective of any pathogen survey is to detect a pathogen in sample given the pathogen is present in the population, we used simulation to calculate the probability of detecting a hypothetical pathogen (i.e., power) giving varying levels of: (1) host-level infection rate, 0.1 to 0.9 by increments of 0.1; (2) conditional organ tissue level infection rate, 0.1 and 0.3 (i.e., organ infection rate of infected hosts); and (3) test sensitivity, 10% and 50%; (4) number of organ surveyed, 1, 3, and 5; (5) number of replicates surveyed, 1–4; and (6) host sample sizes, 5–60. Two thousand replicate datasets then were simulated for all combinations of these parameters and the proportion of the 2000 replicate datasets where the hypothetical pathogen was detected at least once was calculated. The minimum number of hosts needed to detect the pathogen with a power of 80% was determined and graphically summarized. See S1 Information for R code used to replicate analysis.

## Results

### Overview of the data and model fits

Pathogens detected by histological examination of organ tissues included: gill metacercariae, *R*. *salmoninarum*, *N*. *salmincola*, and *P*. *minibicornis*. *Apophallus/*echinostome metacercariae and *N*. *salmincola* metacercariae were detected in gill tissues. Kidney tissues contained detectable levels of *N*. *salmincola*, *R*. *salmoninarum*, and *P*. *minibicornis*. Within the kidney, *P*. *minibicornis* was detected in glomeruli and tubule tissues. *N*. *salmincola* was detected in heart tissues. Only *R*. *salmoninarum* was detected in splenic tissues. Pathogen detections were not perfect (i.e., not detected in every replicate) for all pathogens encountered. In particular, false negatives, identified as detection histories including a combination of 0s and 1s accounted for 4% to 88% of the observed detections (Table 2). Goodness-of-fit checks indicated adequate fit for pathogen-specific occupancy models fit to detection data with p-values that varied from 0.48 to 0.58.

**Table 2:** Frequency of detection combinations for the 26 fish sampled.

		Detection type		
		Non-detection	Imperfect	Perfect
*Apophallus/*echinnostome metacercariae	Gill	17	6	3
*Renibacter salmoninarum*	Kidney	23	23	23
	Liver	25	1	0
	Spleen	25	25	25
*Nanophyetus salmincola*	Gill	0	9	17
	Heart	3	12	11
	Kidney	0	13	13
*Parvicapsula minibicornis*	Glomerulus	4	9	13
	Tubules	5	4	17

Non-detections represent three replicate pathogen non-detections (i.e., 000). Imperfect detections represent possible combinations of replicate pathogen detections and non-detections (i.e., 100, 010, 001, 110, 101, 011). Perfect detections represent three replicate pathogen detections (i.e., 111).

### Fish and organ-level unadjusted pathogen prevalence

Unadjusted pathogen prevalence (α) varied at the host- and organ-levels, and organ-level prevalence was always lower than host-level prevalence (Table 3). Host-level unadjusted prevalence was highest for *N*. *salmincola* (0.96–1.00) and unadjusted prevalence among organs was lower, varying from 0.73 to 0.88. *P*. *minibicornis* host-level unadjusted prevalence varied among replicates from 0.84 to 0.96 and organ-level unadjusted prevalence was lower varying from 0.64 to 0.81. *R*. *salmoninarum* prevalence was lowest among pathogens detected, and among replicate host-level unadjusted prevalence varied from 0.08 to 0.15. *Apophallus/*echinostome metacercariae were only detected in gill tissue and unadjusted prevalence varied among replicates from 0.08 to 0.27.

**Table 3:** Fish and organ-level prevalence (unadjusted for test sensitivity; *a*) for replicated tissue-level detection/non-detection data.

		Replicate		
Pathogen	Level	1	2	3
*Apophallus/*echinostome metacercariae	Gill^a^	0.08	0.27	0.27
*Renibacter salmoninarum*	Fish	0.15	0.08	0.08
	Kidney	0.08	0.08	0.08
	Liver	0.04	0.00	0.00
	Spleen	0.04	0.00	0.00
*Nanophyetus salmincola*	Fish	1.00	1.00	0.96
	Gill	0.84	0.85	0.85
	Heart	0.80	0.81	0.81
	Kidney	0.88	0.73	0.80
*Parvicapsula minibicornis*	Kidney^a^	0.84	0.96	0.96
	Glomerulus	0.64	0.81	0.80
	Tubules	0.68	0.73	0.80

Fish level prevalence represents the aggregation of among organ detections.

^a^ Gill and kidney represent the highest level of detection for *Apophallus/*echinostome metacercariae and *P*. *minibicornis* and therefore fish level prevalence is equal to these values.

### Model estimates

*Sensitivity estimates*.—Model estimated organ tissue-specific sensitivities (*s*) were all less than one and varied among pathogens and organs (Table 4). Estimated sensitivity was lowest for *Apophallus/*echinostome metacercariae in gill tissue (0.54) and greatest for *P*. *minibicornis* in kidney glomeruli (0.91). Sensitivity also varied more among pathogens than for individual pathogens among organs. For instance, sensitivity for *R*. *salmoninarum* in three different organs averaged 0.66 (range 0.63–0.69), whereas *N*. *salmincola* sensitivity averaged 0.85 (range 0.81–0.88). Most sensitivity estimates were relatively precise with the exception of *R*. *salmoninarum* estimates for liver and spleen, which had very large credible intervals (Table 4) likely due to a very low number of detections.

**Table 4:** Estimated organ-specific sensitivity (*s*) and 95% credible intervals for the occupancy model fit to spring Chinook collected from Willamette Hatchery, Oregon.

			95% credible interval	
*Pathogen*	Organ	Estimate	Lower	Upper
*Apophallus/*echinostome metacercariae	Gills	0.544	0.307	0.754
*Renibacter salmoninarum*	Kidney	0.694	0.304	0.947
	Liver	0.626	0.018	0.999
	Spleen	0.660	0.020	0.999
*Nanophyetus salmincola*	Gills	0.841	0.760	0.995
	Heart	0.883	0.796	0.909
	Kidney	0.813	0.719	0.884
*Parvicapsula minibicornis*	Tubules	0.887	0.799	0.948
	Glomerulus	0.912	0.829	0.967

Estimates reported for tissues where pathogens were detected.

Host- and organ-level infection rates

Host- and organ-level infection rates (Ψ, φ) also varied among pathogens detected and ranged from a low of 0.41 for *Apophallus/*echinostome metacercariae to a high of to 0.99 for *N*. *salmincola* (Table 5). In some cases where pathogens infected multiple organs, organ-level infection rates exceeded host-level infection rates, which can occur due to the conditional specification of organ-level infection rates given the host was infected. The unconditional organ-level infection rate are the product of host and organ-level infection rates and are equal to or lower than the host-level infection rate. For example, the probability that heart tissue from a randomly selected fish was infected with *N*. *salmincola* is 0.95•0.99 = 0.94. Conditional organ-level *R*. *salmoninarum* infection rate was highest for the kidney (0.22) and lowest for the liver and spleen, with very imprecise estimates for the latter two organs (Table 5).

**Table 5:** Fish and organ-level infection rates (Ψ, φ) and prevalence estimates (*P*) and credible intervals for the occupancy model fit to spring Chinook collected from Willamette Hatchery, Oakridge, Oregon.

		Infection rate			Prevalence		
			95% credible interval			95% credible interval	
Pathogen	Level	Estimate	Lower	Upper	Estimate	Lower	Upper
*Apophallus/*echinostome metacercariae	Gills^a^	0.414	0.228	0.665	0.401	0.346	0.577
*Renibacter salmoninarum*	Fish	0.564	0.181	0.962	0.567	0.192	0.962
	Kidney	0.219	0.043	0.703	0.123	0.115	0.192
	Liver	0.161	0.010	0.876	0.076	0.038	0.423
	Spleen	0.159	0.011	0.914	0.073	0.038	0.423
*Nanophyetus salmincola*	Fish	0.953	0.862	0.995	1.000	1.000	1.000
	Gills	0.990	0.939	1.000	1.000	1.000	1.000
	Heart	0.901	0.753	0.990	0.906	0.846	0.962
	Kidney	0.990	0.936	1.000	1.000	1.000	1.000
*Parvicapsula minibicornis*	Fish	0.931	0.815	0.989	0.967	0.961	1.000
	Tubules	0.860	0.707	0.964	0.849	0.846	0.885
	Glomerulus	0.823	0.653	0.945	0.809	0.808	0.846

### Prevalence comparison

**Fig 2:**
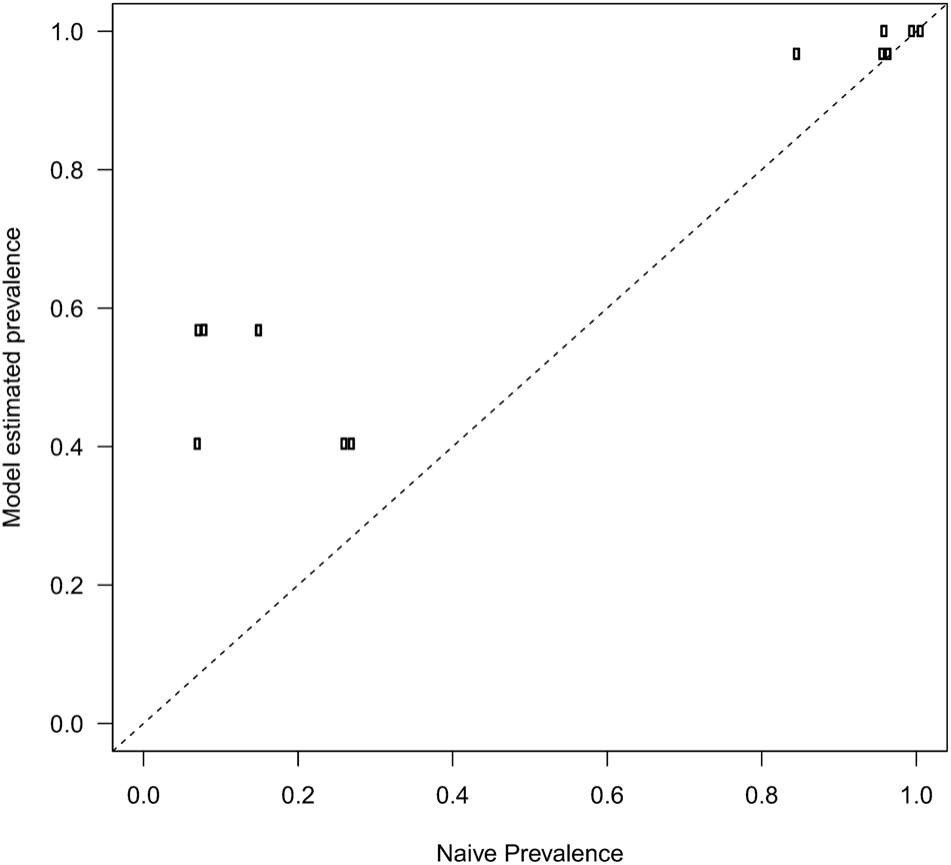
Comparison of host-level model estimated (Ψ) and unadjusted prevalence (α) for pathogens detected. Unadjusted prevalence (α) represents estimates for each replicate (3 per pathogen, 12 estimates total). The dotted line denotes a 1:1 relationship. A small amount of random noise was added due to overplotting. Vertical lines denote 95% credible intervals.

Host-level prevalence unadjusted for test sensitivity (α) was lower than model estimated infection rates (Ψ; Fig. 2). The negative discrepancy decreased as unadjusted prevalence approached one and therefore biases were relatively small for widespread pathogens such as *N*. *salmincola* and *P*. *minibicornis*. The largest discrepancy between model infection rates and unadjusted prevalence was observed for gill metacercariae, which was the only pathogen detected in a single tissue, gill in this case.

### Effects of replicates and hierarchical sampling on pathogen detection

**Fig 3:**
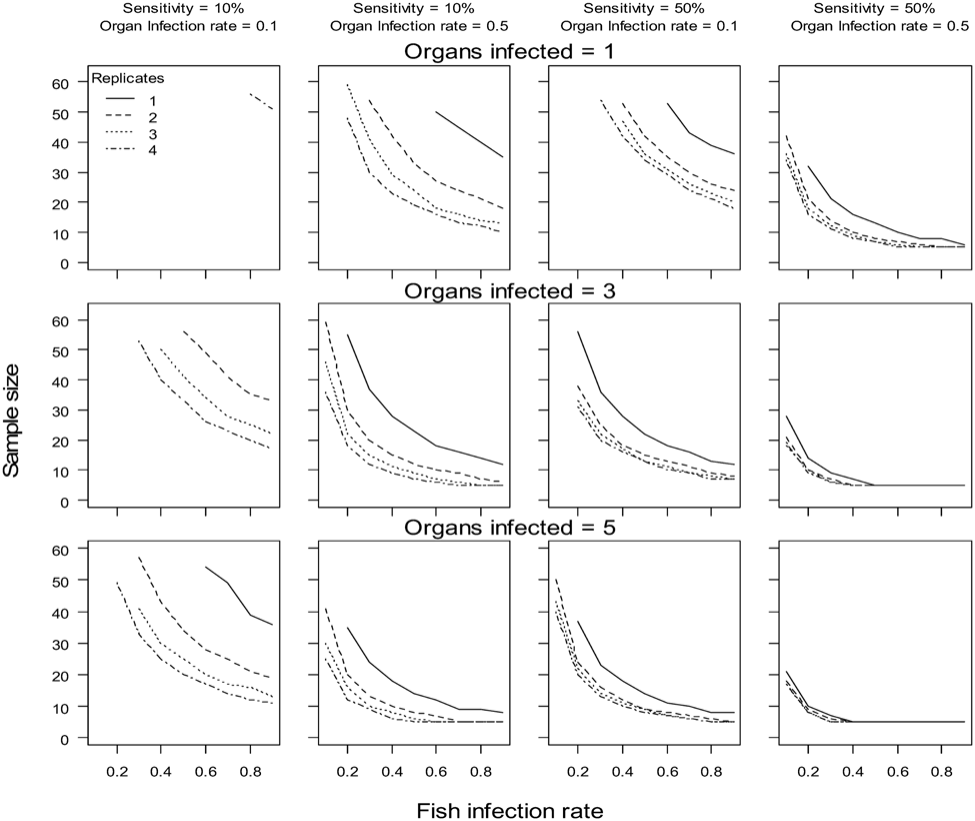
Number of host samples needed to detect a pathogen 80% of the time for varying host-level infection rates (Ψ). Panel rows illustrate the effect of pathogens being searched for in multiple organs and the columns illustrate the effect of varying sensitivity and organ-level infection rate (φ). Organ-level infection rate (φ) and sensitivity (*s*) assumed to be constant among organs in simulations. Sampling sizes for the top left panel required more than 60 and therefore the panel represents the few combinations where the success criteria was met.

The number of host samples required to have an 80% probability of detecting a pathogen in a sample, given it is present, varied among the parameter combinations simulated (Fig. 3). As expected, the required host sample size decreased with increasing host-level and organ-level infection rates. Increasing the number of within organ replicates decreased the host sample size required to detect the pathogen in the sample. This reduction in host sample size was especially apparent for 2 or more replicates when sensitivities were low (10%). However, gains in terms of host sample size reduction with increasing number of replicates diminished with increasing number of organs surveyed.

## Discussion

We developed an approach to use hierarchically structured pathogen detection data from imperfect diagnostic tests to estimate test sensitivity and use that information to achieve unbiased estimation of pathogen infection rate in host populations and among organs within infected hosts. The approach provided a potentially cost-efficient means to improve the effectiveness of lower sensitivity diagnostic tests, such as histology, which is advantageous when pathogen infections are unknown prior to examination [17]. Histology also provides information on detections that are biologically meaningful, representing organ or tissue damage at a histological level, which may be preferable to highly sensitive molecular diagnostic tests that can detect pathogen presence, but disease status may be uncertain (i.e., pathogen is present but not causing disease or pathogen may be dead).

All pathogens found in this study were detected imperfectly (i.e., present in host and organ but not detected in every tissue sample). Imperfect pathogen detection within infected hosts and organs can arise as a result of pathogen distribution and abundance. Non-detection can occur if tissues are sampled prior to pathogen dispersal through the host and subsequent colonization of organ tissues and due to localized areas of focal infection that may not be contained in histological sections. In this case, once pathogens infect a host, detection depends on the pathogens dispersing, infecting, and distributing within host organ tissues. For example, proliferative pathogens, such as *R*. *salmoninarum* are likely distributed in relation to the area of initial infection in early stages. Detection of the pathogen will be difficult unless the area of initial infection is contained in the sampled tissue. Related to infection distribution, pathogen detection will generally be related to intensity of infection. In particular, as pathogen intensity increases due to proliferation or continuous colonization of host tissues, the probability of encountering and detecting a pathogen generally increases. Test sensitivity is likely to be low for sublinical infections because pathogen intensities are low and their distribution is likely to be focalized to few locations and therefore difficult to detect.

Host-level unadjusted prevalence was always higher than organ-level unadjusted prevalence, due to the inclusion of multiple organ-level observations where a pathogen was detected in one organ but not others. Sampling multiple organs within an individual constitutes an ad hoc approach to account for imperfect detection and inform individual level infection status, similar to spatial resampling [44–46]. In other words, evaluating multiple organs and aggregating detections to the host provided multiple opportunities to detect the pathogen. Discrepancies between host-level unadjusted and model estimated infection rates were lower for pathogens that infected multiple organs, such as *N*. *salmincola*, due to these additional detections from multiple organs. Because sampling multiple organ tissues provided additional opportunities to detect a pathogen, discrepancies between prevalence estimates was the largest for gill metacercariae, which were only detected in gill tissue.

The occupancy model used in this study provided estimates of diagnostic test sensitivity. Pathogen-specific test sensitivities estimates reported in our earlier study [17] and this study were similar for *N*. *salmincola*, but higher for *R*. *salmoninarum*. This is not unexpected as sensitivity is likely dependent on pathogen abundance and distribution within tissues and will vary among studies, unless pathogen abundances and distribution are similar within tissues. Therefore, the discrepancy with *R*. *salmoninarum* sensitivity estimates reported in Kent et al. [17] is likely due to pathogen and associated lesion abundance and distribution differences within organ tissues. The samples from Kent et al. [17] include adult Chinook salmon from rivers, including moribund fish. These likely had higher *R*. *salmoninarum* levels relative to the source of hatchery fish used in this study, which received prophylactic antibiotic injections. The relatively few detections and high variability of sensitivity estimates for *R*. *salmoninarum* in the present study were likely influenced by relatively low *R*. *salmoninarum* abundances in individuals. Another explanation for variability between sensitivity estimates is due to the differences in how sensitivity was calculated. Kent et al. [17] used enzyme linked immunosorbent assay (ELISA) to determine whether *R*. *salmoninarum* was present in the host. While the highly sensitivity ELISA can readily detect *R*. *salmoninarum* presence, it does not necessarily translate to infection as observed by histology because this test detects bacterial proteins that may persist long after viable infections have resolved [47]. Because we estimated organ tissue-specific sensitivity for histology by repeated detections of infection using multiple tissue samples, it provided more realistic sensitivity estimates relative to comparing infection detections to pathogen presence, which may not be present at sufficient levels to cause clinical infection.

Organ-level infection rate estimates were conditional on the host infection state (infected or not) and represent the probability that an organ is infected given the host is infected. Therefore, these estimates provide useful information regarding pathogen specificity among organs and can inform sample designs and pathogen dynamics. For example, the estimated *R*. *salmoninarum* organ-level infection rates were greatest for kidney tissues, indicating that if an individual was infected, the pathogen is most likely found in the kidney. This makes biological sense because *R*. *salmoninarum* frequently occurs in the kidney and is the causative agent of bacterial kidney disease. Similarly, organ-specific conditional infection rates can provide insight into pathogen tissue preferences, where differences among conditional organ-level infection rates can provide evidence for hypothesized pathogen specific tissue preferences. These organ-level infection rates also provide information on what organs to sample if resources are limiting. For example, given conditional organ-level infection rates, *N*. *salmincola* was most likely found in gill and kidney tissues relative to heart. This information can help to inform sample design and evaluate tradeoffs among designs when resources are limiting and sampling of multiple organs is not feasible.

Sampling enough individual hosts to have sufficient power to detect a pathogen in a population sample, given it is present, is necessary for potential analysis of host-pathogen interactions and establishing sampling designs. Surveying multiple organs and using replicate tissue sampling within those organs increased the likelihood of encountering a pathogen and provided at least 2 benefits over traditional single samples. First, replicate sampling allowed for the estimation of sensitivity and unbiased infection rates and associated uncertainty. Second, by accounting for test sensitivity using within host replicate tissue samples, fewer individuals need to be handled, providing a beneficial reduction of animal use, an essential component of ethical animal use [24]. This effect was illustrated in the simulation analysis, where increasing the number of organs surveyed (assuming the pathogen infects multiple organs) and tissue replicates within host reduced the host sample size required to achieve a power of 80%. Additional general guiding rules that emerged from the simulation analysis indicated that the effect of increasing within organ replicates was minimal when the pathogen can be detected in multiple organs. However, this result does not imply that within organ replicates are not an important component of the sample design. Within organ replicates are critical for accurately estimating organ-level infection rates and reducing associated uncertainties [48]. Additional studies have demonstrated the tradeoffs involved with allocating efforts and provide guidance on occupancy-detection type survey designs [49–51]

Encountering and detecting a pathogen within host organ tissue is related, in part, to pathogen abundance and distribution and therefore sensitivities can vary with intensity of infection. Infection levels also will vary among the individuals sampled and this variation can influence sensitivity estimates. In particular, aggregation of high pathogen levels in few individuals is a common phenomenon encountered when sampling host populations, particularly with macroparasites [52]. Accounting for variation in pathogen abundance will improve estimates of infection rates by accounting for heterogeneous sensitivities. The analytical framework we used can account for this by predicting sensitivity as a function of auxiliary data, if those data are available. In other words, this approach could model sensitivity as function of additional measures of pathogen abundance. For example, the use of quantitative assays such as ELISA or qPCR that provide quantitative measures associated with pathogen abundance could be used to account for variability of pathogen abundances among hosts. Similarly, quantitative metacercariae counts for worm pathogens (e.g., *N*. *salmincola*) performed from wet mount preparations of pre-weighed tissue samples [18,53] could be used to provide additional quantitative information to predict the probability of detecting the pathogen given pathogen abundance or density.

Using models to predict infection probabilities provides a formal approach to evaluate hypotheses. For example, *R*. *salmoninarum* infections are well recognized in kidney tissues, and so it could be hypothesized that infection of organs other than the kidney may be dependent on kidney infection. This hypothesis can be evaluated by predicting organ-level infection rate as a function of kidney status (i.e., infected, uninfected). Similarly, the effects of host-level information can be used to evaluate hypotheses relating to host-level infection rates. For example, host-level infection rates could be predicted as a function of host weight or length to evaluate the potential for size dependent effects on host-level infection rates. Similarly, the relation between infection rate and host-specific characteristics such as sex, age, and condition also could be evaluated using this approach. Thus, the approach used in this study provides a formal analytical method to incorporate information from multiple diagnostic tests to additionally inform estimates of infection rate and test sensitivity and potentially evaluate hypotheses.

Precise and unbiased prevalence estimates with diagnostic tests are a crucial component of monitoring, research management, or quarantine efforts [33,54,55]. The occupancy-detection estimation approach developed in this study provides a formal method to utilize diagnostic tests with varying sensitivity to analyze pathogen detection data and avoid negative biases due to imperfect detections, which has received recent attention as being an important component of biological surveys [56]. It also provides a framework for analyzing pathogen detection data from multiple organ tissue samples within hosts. This approach is not limited to evaluating pathogens and could use other diagnostic endpoints, such as histological manifestations (e.g., neoplasms) or to evaluate contaminant or toxicant presence in multiple organ tissues. Additionally, the approach also could be extended to evaluate pathogen assemblages and co-occurrence by simultaneous analysis in a multi-pathogen occupancy models similar to those used in ecological studies [57]. Such an approach appears especially appropriate when considering the parasite community as potential population regulatory factors for wild hosts.

## Supporting Information

S1 Information Model Code. WinBUGS code provided to replicate analysis and R code to simulate data for model verification.
(PDF)
