# Supplementary material for: Occupancy Modeling for Improved Accuracy and Understanding of Pathogen Prevalence and Dynamics
Source: PLoS One. 2015 Mar 4;10(3):e0116605. doi: 10.1371/journal.pone.0116605 (PMC4349882; doi:10.1371/journal.pone.0116605)
Supplement: S1 Information — WinBUGS code provided to replicate analysis and R code to simulate data for model verification. (PDF) [file pone.0116605.s001.pdf]

Supporting information 1. R and WinBUGS code provided to replicate analysis.

The following is the base WinBUGS model used in analysis and R code for simulating hypothetical pathogen detections.

Base WinBUGS code

```
model{
    for (i in 1:nfish) {
        logit(fish.psi0[i]) <- alpha0
        fish.psi[i] <- max(1.00000E-05, min(0.99999, fish.psi0[i]))
        lat.fish[i] ~ dbern(fish.psi[i])
        for (j in 1:no.orgs) {
            logit(organ.psi0[i, j]) <- beta[j]
            organ.psi[i, j]<-max(1.00000E-05,min(0.99999,organ.psi0[i,j]))*lat.fish[i]
            lat.org[i, j] ~ dbern(organ.psi[i, j])
            lat.org2[i, j] <- lat.org[i, j] + 1
            for (h in 1:3) {
                logit(theta[i, j, h, 2]) <- gamma[j]
                theta2[i, j, h, 1] <- 0.00000E+00
                theta2[i, j, h, 2] <- max(1.00000E-05, min(0.99999,theta[i, j, h, 2]))
                y[i, j, h] ~ dbin(theta2[i, j, h, lat.org2[i,
                  j]], 1)
            }
        }
    }
    for (i in 1:nfish) {
        for (j in 1:no.orgs) {
            for (h in 1:3) {
                expected[i, j, h] <- theta2[i, j, h, lat.org2[i,j]]
                resid[i, j, h] <- y[i, j, h] - expected[i, j,h]
                residSquared[i, j, h] <- pow(resid[i, j, h],2)
                y.sim[i, j, h] ~ dbin(theta2[i, j, h, lat.org2[i,j]], 1)
                resid.sim[i, j, h] <- y.sim[i, j, h] - expected[i,j, h]
                residSquared.sim[i, j, h] <- pow(resid.sim[i,j, h], 2)
            }
```

```
        }
    }
    rss <- sum(residSquared[, , ])
    rss_sim <- sum(residSquared.sim[, , ])
    for (i in 1:5) {
        prev_org[i] <- sum(lat.org[, i])/nfish
    }
    prev <- sum(lat.fish[])/nfish
    logit(pred.fish) <- alpha0
    for (o in 1:5) {
        logit(pred_organ_psi[o]) <- beta[o]
        logit(pred_organ_p[o]) <- gamma[o]
    }
    alpha0 ~ dnorm(0.00000E+00, 0.37)
    beta0 ~ dnorm(0.00000E+00, 0.37)
    gamma0 ~ dnorm(0.00000E+00, 0.37)
    for (z in 1:no.orgs) {
        beta[z] ~ dnorm(beta0, beta.tau)
        gamma[z] ~ dnorm(gamma0, gamma.tau)
    }
    beta.tau <- pow(beta.ss, -2)
    beta.ss ~ dunif(0.00000E+00, 6)
    gamma.tau <- pow(gamma.ss, -2)
    gamma.ss ~ dunif(0.00000E+00, 6)
}
```

R code for simulating hypothetical pathogen detections

```
# FUNCTION TO SIMULATE DATA
sim_dat<- function(psi_fish,psi_organ,p_organ,n_fish,reps)
     { # returns an array of 0 and 1 formatted for base model
     psi_f<- psi_fish
     psi_o<- psi_organ
     p<- p_organ
     n<- n_fish
     reps<- reps
     z_fish<- rbinom(n,1,psi_f)
     z<- matrix(0, nrow=n, ncol=length(p))
     for(i in 1:length(p))
          {
          z[,i]<- rbinom(n,1, psi_o[i]*z_fish)
          }
     y<- array(0,dim=c(n,length(psi_o),reps))
     for(r in 1:reps)
          {
          for(organ in 1:length(psi_o))
               {
               y[,organ,r]<- rbinom(n,1,z[,organ]*p[organ])
               }
          }
     out<-list(y=y,prev_true = mean(z_fish))
     return(out)
     }
# END FUNCTION

N<- seq(5,60,1)
norgs<- c(1,2,3,4,5)
psi_f<- seq(0.1, 0.9,0.1) #seq(0.1,0.9,0.1)
psi_o<- c(0.1, 0.5, 0.9) #seq(0.1,0.9,0.1)
p_o<- c(0.1, 0.5) # seq(0.1,0.9,0.1)
reps<- c(1:5)
out<- expand.grid(N=N,
norgs=norgs,psi_f=psi_f,reps=reps,psi_o=psi_o, p_o=p_o)
out$typeII<- 0

# DO SIMUATION FOR PARAMETER COMBINATIONS
     for(i in 1:nrow(out))
          {
          yyy<- sapply(c(1:2000),function(x){
          dat<- sim_dat(psi_fish=out$psi_f[i],
          psi_organ=rep(out$psi_o[i], out$norgs[i]),
```

```
p_organ=rep(out$p_o[i], out$norgs[i]),
n_fish = out$N[i],
reps=out$reps[i])
return(mean(dat$y))
})
out$typeII[i]<- mean(ifelse(yyy>0,1,0))
}
```

S2. Analysis data containing data used for analysis. The parasite codes are as follows: *Apophallus/*echinostome: *Ae, Renibacter salmoninarum: Rs, Nanophyetus salmincola: Ns, Parvicapsula minibicornis: Pm*

id	organ	parasite	rep	pres
WH 26	kid_glo	Pm	1	0
WH21	kid_glo	Pm	1	1
WH 27	kid_glo	Pm	1	1
WH 24	kid_glo	Pm	1	0
WH 22	kid_glo	Pm	1	0
WH 440	kid_glo	Pm	1	0
WH 18	kid_glo	Pm	1	1
WH 19	kid_glo	Pm	1	1
WH 438	kid_glo	Pm	1	1
WH 29	kid_glo	Pm	1	1
WH 23	kid_glo	Pm	1	1
WH 28	kid_glo	Pm	1	1
WH 425	kid_glo	Pm	1	1
WH 31	kid_glo	Pm	1	0
WH 390	kid_glo	Pm	1	0
WH 381	kid_glo	Pm	1	1
WH 100	kid_glo	Pm	1	1
WH 107	kid_glo	Pm	1	1
WH 96F	kid_glo	Pm	1	0
WH F104	kid_glo	Pm	1	1
WH 25	kid_glo	Pm	1	1
WHF93	kid_glo	Pm	1	0
WH32	kid_glo	Pm	1	0
WH 429	kid_glo	Pm	1	1
WH 20	kid_glo	Pm	1	1
WH 26	kid_tub	Pm	1	1
WH21	kid_tub	Pm	1	0
WH 27	kid_tub	Pm	1	1
WH 24	kid_tub	Pm	1	1
WH 22	kid_tub	Pm	1	1
WH 440	kid_tub	Pm	1	0
WH 18	kid_tub	Pm	1	0
WH 19	kid_tub	Pm	1	1
WH 438	kid_tub	Pm	1	0
WH 29	kid_tub	Pm	1	1
WH 23	kid_tub	Pm	1	1
WH 28	kid_tub	Pm	1	1
WH 425	kid_tub	Pm	1	1
WH 31	kid_tub	Pm	1	0

id	organ	parasite	rep	pres
WH 390	kid_tub	Pm	1	0
WH 381	kid_tub	Pm	1	1
WH 100	kid_tub	Pm	1	1
WH 107	kid_tub	Pm	1	1
WH 96F	kid_tub	Pm	1	1
WH F104	kid_tub	Pm	1	1
WH 25	kid_tub	Pm	1	1
WHF93	kid_tub	Pm	1	0
WH32	kid_tub	Pm	1	1
WH 429	kid_tub	Pm	1	0
WH 20	kid_tub	Pm	1	1
WH 26	gill	AE	1	0
WH21	gill	AE	1	0
WH 27	gill	AE	1	1
WH 24	gill	AE	1	0
WH 22	gill	AE	1	0
WH 440	gill	AE	1	0
WH 18	gill	AE	1	0
WH 19	gill	AE	1	0
WH 438	gill	AE	1	0
WH 29	gill	AE	1	0
WH 23	gill	AE	1	0
WH 28	gill	AE	1	0
WH 425	gill	AE	1	0
WH 31	gill	AE	1	0
WH 390	gill	AE	1	0
WH 381	gill	AE	1	0
WH 100	gill	AE	1	0
WH 107	gill	AE	1	0
WH 96F	gill	AE	1	0
WH F104	gill	AE	1	0
WH 25	gill	AE	1	1
WHF93	gill	AE	1	0
WH32	gill	AE	1	0
WH 429	gill	AE	1	0
WH 20	gill	AE	1	0
WH 26	kid	RS	1	0
WH21	kid	RS	1	0
WH 27	kid	RS	1	0
WH 24	kid	RS	1	0
WH 22	kid	RS	1	0
WH 440	kid	RS	1	0
WH 18	kid	RS	1	0

id	organ	parasite	rep	pres
WH 19	kid	RS	1	0
WH 438	kid	RS	1	0
WH 29	kid	RS	1	0
WH 23	kid	RS	1	1
WH 28	kid	RS	1	0
WH 425	kid	RS	1	0
WH 31	kid	RS	1	0
WH 390	kid	RS	1	0
WH 381	kid	RS	1	0
WH 100	kid	RS	1	0
WH 107	kid	RS	1	0
WH 96F	kid	RS	1	0
WH F104	kid	RS	1	0
WH 25	kid	RS	1	0
WHF93	kid	RS	1	0
WH32	kid	RS	1	1
WH 429	kid	RS	1	0
WH 20	kid	RS	1	0
WH 26	liv	RS	1	0
WH21	liv	RS	1	0
WH 27	liv	RS	1	0
WH 24	liv	RS	1	0
WH 22	liv	RS	1	0
WH 440	liv	RS	1	0
WH 18	liv	RS	1	0
WH 19	liv	RS	1	0
WH 438	liv	RS	1	0
WH 29	liv	RS	1	0
WH 23	liv	RS	1	0
WH 28	liv	RS	1	0
WH 425	liv	RS	1	0
WH 31	liv	RS	1	0
WH 390	liv	RS	1	0
WH 381	liv	RS	1	0
WH 100	liv	RS	1	1
WH 107	liv	RS	1	0
WH 96F	liv	RS	1	0
WH F104	liv	RS	1	0
WH 25	liv	RS	1	0
WHF93	liv	RS	1	0
WH32	liv	RS	1	0
WH105	liv	RS	1	0
WH 429	liv	RS	1	0

id	organ	parasite	rep	pres
WH 20	liv	RS	1	0
WH 26	spl	RS	1	0
WH21	spl	RS	1	0
WH 27	spl	RS	1	0
WH 24	spl	RS	1	0
WH 22	spl	RS	1	0
WH 440	spl	RS	1	0
WH 18	spl	RS	1	0
WH 19	spl	RS	1	0
WH 438	spl	RS	1	0
WH 29	spl	RS	1	0
WH 23	spl	RS	1	0
WH 28	spl	RS	1	0
WH 425	spl	RS	1	0
WH 31	spl	RS	1	0
WH 390	spl	RS	1	0
WH 381	spl	RS	1	0
WH 100	spl	RS	1	0
WH 107	spl	RS	1	0
WH 96F	spl	RS	1	1
WH F104	spl	RS	1	0
WH 25	spl	RS	1	0
WHF93	spl	RS	1	0
WH32	spl	RS	1	0
WH105	spl	RS	1	0
WH 429	spl	RS	1	0
WH 20	spl	RS	1	0
WH 26	gill	Nano	1	0
WH21	gill	Nano	1	1
WH 27	gill	Nano	1	0
WH 24	gill	Nano	1	1
WH 22	gill	Nano	1	1
WH 440	gill	Nano	1	1
WH 18	gill	Nano	1	0
WH 19	gill	Nano	1	1
WH 29	gill	Nano	1	1
WH 23	gill	Nano	1	1
WH 28	gill	Nano	1	0
WH 425	gill	Nano	1	1
WH 31	gill	Nano	1	1
WH 390	gill	Nano	1	1
WH 381	gill	Nano	1	1
WH 100	gill	Nano	1	1

id	organ	parasite	rep	pres
WH 107	gill	Nano	1	1
WH 96F	gill	Nano	1	1
WH F104	gill	Nano	1	1
WH 25	gill	Nano	1	1
WHF93	gill	Nano	1	1
WH32	gill	Nano	1	1
WH105	gill	Nano	1	1
WH 429	gill	Nano	1	1
WH 20	gill	Nano	1	1
WH 26	kid	Nano	1	0
WH21	kid	Nano	1	1
WH 27	kid	Nano	1	1
WH 24	kid	Nano	1	1
WH 22	kid	Nano	1	1
WH 440	kid	Nano	1	0
WH 18	kid	Nano	1	1
WH 19	kid	Nano	1	0
WH 438	kid	Nano	1	1
WH 29	kid	Nano	1	1
WH 23	kid	Nano	1	1
WH 28	kid	Nano	1	1
WH 425	kid	Nano	1	1
WH 31	kid	Nano	1	1
WH 390	kid	Nano	1	1
WH 381	kid	Nano	1	1
WH 100	kid	Nano	1	1
WH 107	kid	Nano	1	1
WH 96F	kid	Nano	1	1
WH F104	kid	Nano	1	1
WH 25	kid	Nano	1	1
WHF93	kid	Nano	1	1
WH32	kid	Nano	1	1
WH 429	kid	Nano	1	1
WH 20	kid	Nano	1	1
WH 26	hrt	Nano	1	1
WH21	hrt	Nano	1	0
WH 27	hrt	Nano	1	1
WH 24	hrt	Nano	1	0
WH 22	hrt	Nano	1	1
WH 440	hrt	Nano	1	1
WH 18	hrt	Nano	1	1
WH 19	hrt	Nano	1	1
WH 29	hrt	Nano	1	1

id	organ	parasite	rep	pres
WH 23	hrt	Nano	1	1
WH 28	hrt	Nano	1	0
WH 425	hrt	Nano	1	1
WH 31	hrt	Nano	1	1
WH 390	hrt	Nano	1	1
WH 381	hrt	Nano	1	0
WH 100	hrt	Nano	1	1
WH 107	hrt	Nano	1	1
WH 96F	hrt	Nano	1	1
WH F104	hrt	Nano	1	1
WH 25	hrt	Nano	1	1
WHF93	hrt	Nano	1	1
WH32	hrt	Nano	1	1
WH105	hrt	Nano	1	1
WH 429	hrt	Nano	1	0
WH 20	hrt	Nano	1	1
WH 26	kid	Furn	1	0
WH21	kid	Furn	1	0
WH 27	kid	Furn	1	0
WH 24	kid	Furn	1	0
WH 22	kid	Furn	1	0
WH 440	kid	Furn	1	0
WH 18	kid	Furn	1	0
WH 19	kid	Furn	1	0
WH 438	kid	Furn	1	0
WH 29	kid	Furn	1	0
WH 23	kid	Furn	1	0
WH 28	kid	Furn	1	0
WH 425	kid	Furn	1	0
WH 31	kid	Furn	1	0
WH 390	kid	Furn	1	0
WH 381	kid	Furn	1	0
WH 100	kid	Furn	1	0
WH 107	kid	Furn	1	0
WH 96F	kid	Furn	1	0
WH F104	kid	Furn	1	0
WH 25	kid	Furn	1	0
WHF93	kid	Furn	1	0
WH32	kid	Furn	1	0
WH105	kid	Furn	1	0
WH 429	kid	Furn	1	0
WH 20	kid	Furn	1	0
WH 26	hrt	Furn	1	0

id	organ	parasite	rep	pres
WH21	hrt	Furn	1	0
WH 27	hrt	Furn	1	0
WH 24	hrt	Furn	1	0
WH 22	hrt	Furn	1	0
WH 440	hrt	Furn	1	0
WH 18	hrt	Furn	1	0
WH 19	hrt	Furn	1	0
WH 438	hrt	Furn	1	0
WH 29	hrt	Furn	1	0
WH 23	hrt	Furn	1	0
WH 28	hrt	Furn	1	0
WH 425	hrt	Furn	1	0
WH 31	hrt	Furn	1	0
WH 390	hrt	Furn	1	0
WH 381	hrt	Furn	1	0
WH 100	hrt	Furn	1	0
WH 107	hrt	Furn	1	0
WH 96F	hrt	Furn	1	0
WH F104	hrt	Furn	1	0
WH 25	hrt	Furn	1	0
WHF93	hrt	Furn	1	0
WH32	hrt	Furn	1	0
WH105	hrt	Furn	1	0
WH 429	hrt	Furn	1	0
WH 20	hrt	Furn	1	0
WH 26	spl	Furn	1	0
WH21	spl	Furn	1	0
WH 27	spl	Furn	1	0
WH 24	spl	Furn	1	0
WH 22	spl	Furn	1	0
WH 440	spl	Furn	1	0
WH 18	spl	Furn	1	0
WH 19	spl	Furn	1	0
WH 438	spl	Furn	1	0
WH 29	spl	Furn	1	0
WH 23	spl	Furn	1	0
WH 28	spl	Furn	1	0
WH 425	spl	Furn	1	0
WH 31	spl	Furn	1	0
WH 390	spl	Furn	1	0
WH 381	spl	Furn	1	0
WH 100	spl	Furn	1	0
WH 107	spl	Furn	1	0

id	organ	parasite	rep	pres
WH 96F	spl	Furn	1	0
WH F104	spl	Furn	1	0
WH 25	spl	Furn	1	0
WHF93	spl	Furn	1	0
WH32	spl	Furn	1	0
WH105	spl	Furn	1	0
WH 429	spl	Furn	1	0
WH 20	spl	Furn	1	0
WH 26	liv	Furn	1	0
WH21	liv	Furn	1	0
WH 27	liv	Furn	1	0
WH 24	liv	Furn	1	0
WH 22	liv	Furn	1	0
WH 440	liv	Furn	1	0
WH 18	liv	Furn	1	0
WH 19	liv	Furn	1	0
WH 438	liv	Furn	1	0
WH 29	liv	Furn	1	0
WH 23	liv	Furn	1	0
WH 28	liv	Furn	1	0
WH 425	liv	Furn	1	0
WH 31	liv	Furn	1	0
WH 390	liv	Furn	1	0
WH 381	liv	Furn	1	0
WH 100	liv	Furn	1	0
WH 107	liv	Furn	1	0
WH 96F	liv	Furn	1	0
WH F104	liv	Furn	1	0
WH 25	liv	Furn	1	0
WHF93	liv	Furn	1	0
WH32	liv	Furn	1	0
WH105	liv	Furn	1	0
WH 429	liv	Furn	1	0
WH 20	liv	Furn	1	0
WH 26	gill	Furn	1	0
WH21	gill	Furn	1	0
WH 27	gill	Furn	1	0
WH 24	gill	Furn	1	0
WH 22	gill	Furn	1	0
WH 440	gill	Furn	1	0
WH 18	gill	Furn	1	0
WH 19	gill	Furn	1	0
WH 438	gill	Furn	1	0

id	organ	parasite	rep	pres
WH 29	gill	Furn	1	0
WH 23	gill	Furn	1	0
WH 28	gill	Furn	1	0
WH 425	gill	Furn	1	0
WH 31	gill	Furn	1	0
WH 390	gill	Furn	1	0
WH 381	gill	Furn	1	0
WH 100	gill	Furn	1	0
WH 107	gill	Furn	1	0
WH 96F	gill	Furn	1	0
WH F104	gill	Furn	1	0
WH 25	gill	Furn	1	0
WHF93	gill	Furn	1	0
WH32	gill	Furn	1	0
WH105	gill	Furn	1	0
WH 429	gill	Furn	1	0
WH 20	gill	Furn	1	0
WH 26	kid_glo	Pm	2	1
WH21	kid_glo	Pm	2	1
WH 27	kid_glo	Pm	2	1
WH 24	kid_glo	Pm	2	1
WH 22	kid_glo	Pm	2	0
WH 440	kid_glo	Pm	2	1
WH 18	kid_glo	Pm	2	1
WH 19	kid_glo	Pm	2	1
WH 438	kid_glo	Pm	2	1
WH 29	kid_glo	Pm	2	1
WH 23	kid_glo	Pm	2	1
WH 28	kid_glo	Pm	2	1
WH 425	kid_glo	Pm	2	1
WH 31	kid_glo	Pm	2	1
WH 390	kid_glo	Pm	2	1
WH 381	kid_glo	Pm	2	0
WH 100	kid_glo	Pm	2	1
WH 107	kid_glo	Pm	2	1
WH 96F	kid_glo	Pm	2	0
WH F104	kid_glo	Pm	2	1
WH 25	kid_glo	Pm	2	1
WHF93	kid_glo	Pm	2	0
WH32	kid_glo	Pm	2	0
WH105	kid_glo	Pm	2	1
WH 429	kid_glo	Pm	2	1
WH 20	kid_glo	Pm	2	1

id	organ	parasite	rep	pres
WH 26	kid_tub	Pm	2	1
WH21	kid_tub	Pm	2	0
WH 27	kid_tub	Pm	2	1
WH 24	kid_tub	Pm	2	1
WH 22	kid_tub	Pm	2	1
WH 440	kid_tub	Pm	2	0
WH 18	kid_tub	Pm	2	1
WH 19	kid_tub	Pm	2	1
WH 438	kid_tub	Pm	2	0
WH 29	kid_tub	Pm	2	1
WH 23	kid_tub	Pm	2	1
WH 28	kid_tub	Pm	2	1
WH 425	kid_tub	Pm	2	1
WH 31	kid_tub	Pm	2	0
WH 390	kid_tub	Pm	2	0
WH 381	kid_tub	Pm	2	1
WH 100	kid_tub	Pm	2	1
WH 107	kid_tub	Pm	2	1
WH 96F	kid_tub	Pm	2	1
WH F104	kid_tub	Pm	2	1
WH 25	kid_tub	Pm	2	1
WHF93	kid_tub	Pm	2	0
WH32	kid_tub	Pm	2	1
WH105	kid_tub	Pm	2	1
WH 429	kid_tub	Pm	2	0
WH 20	kid_tub	Pm	2	1
WH 26	gill	AE	2	1
WH21	gill	AE	2	0
WH 27	gill	AE	2	1
WH 24	gill	AE	2	0
WH 22	gill	AE	2	1
WH 440	gill	AE	2	0
WH 18	gill	AE	2	0
WH 19	gill	AE	2	0
WH 438	gill	AE	2	1
WH 29	gill	AE	2	0
WH 23	gill	AE	2	0
WH 28	gill	AE	2	0
WH 425	gill	AE	2	0
WH 31	gill	AE	2	0
WH 390	gill	AE	2	0
WH 381	gill	AE	2	0
WH 100	gill	AE	2	0

id	organ	parasite	rep	pres
WH 107	gill	AE	2	0
WH 96F	gill	AE	2	0
WH F104	gill	AE	2	0
WH 25	gill	AE	2	1
WHF93	gill	AE	2	0
WH32	gill	AE	2	0
WH105	gill	AE	2	1
WH 429	gill	AE	2	0
WH 20	gill	AE	2	1
WH 26	kid	RS	2	0
WH21	kid	RS	2	0
WH 27	kid	RS	2	0
WH 24	kid	RS	2	0
WH 22	kid	RS	2	0
WH 440	kid	RS	2	0
WH 18	kid	RS	2	0
WH 19	kid	RS	2	0
WH 438	kid	RS	2	0
WH 29	kid	RS	2	0
WH 23	kid	RS	2	0
WH 28	kid	RS	2	0
WH 425	kid	RS	2	0
WH 31	kid	RS	2	0
WH 390	kid	RS	2	0
WH 381	kid	RS	2	0
WH 100	kid	RS	2	0
WH 107	kid	RS	2	0
WH 96F	kid	RS	2	0
WH F104	kid	RS	2	0
WH 25	kid	RS	2	0
WHF93	kid	RS	2	0
WH32	kid	RS	2	1
WH105	kid	RS	2	1
WH 429	kid	RS	2	0
WH 20	kid	RS	2	0
WH 26	liv	RS	2	0
WH21	liv	RS	2	0
WH 27	liv	RS	2	0
WH 24	liv	RS	2	0
WH 22	liv	RS	2	0
WH 440	liv	RS	2	0
WH 18	liv	RS	2	0
WH 19	liv	RS	2	0

id	organ	parasite	rep	pres
WH 438	liv	RS	2	0
WH 29	liv	RS	2	0
WH 23	liv	RS	2	0
WH 28	liv	RS	2	0
WH 425	liv	RS	2	0
WH 31	liv	RS	2	0
WH 390	liv	RS	2	0
WH 381	liv	RS	2	0
WH 100	liv	RS	2	0
WH 107	liv	RS	2	0
WH 96F	liv	RS	2	0
WH F104	liv	RS	2	0
WH 25	liv	RS	2	0
WHF93	liv	RS	2	0
WH32	liv	RS	2	0
WH105	liv	RS	2	0
WH 20	liv	RS	2	0
WH 26	spl	RS	2	0
WH21	spl	RS	2	0
WH 27	spl	RS	2	0
WH 24	spl	RS	2	0
WH 22	spl	RS	2	0
WH 440	spl	RS	2	0
WH 18	spl	RS	2	0
WH 19	spl	RS	2	0
WH 438	spl	RS	2	0
WH 29	spl	RS	2	0
WH 23	spl	RS	2	0
WH 28	spl	RS	2	0
WH 425	spl	RS	2	0
WH 31	spl	RS	2	0
WH 390	spl	RS	2	0
WH 381	spl	RS	2	0
WH 100	spl	RS	2	0
WH 107	spl	RS	2	0
WH 96F	spl	RS	2	0
WH F104	spl	RS	2	0
WH 25	spl	RS	2	0
WHF93	spl	RS	2	0
WH32	spl	RS	2	0
WH105	spl	RS	2	0
WH 20	spl	RS	2	0
WH 26	gill	Nano	2	0

id	organ	parasite	rep	pres
WH21	gill	Nano	2	0
WH 27	gill	Nano	2	0
WH 24	gill	Nano	2	1
WH 22	gill	Nano	2	0
WH 440	gill	Nano	2	1
WH 18	gill	Nano	2	1
WH 19	gill	Nano	2	1
WH 438	gill	Nano	2	1
WH 29	gill	Nano	2	1
WH 23	gill	Nano	2	1
WH 28	gill	Nano	2	1
WH 425	gill	Nano	2	1
WH 31	gill	Nano	2	1
WH 390	gill	Nano	2	1
WH 381	gill	Nano	2	1
WH 100	gill	Nano	2	1
WH 107	gill	Nano	2	1
WH 96F	gill	Nano	2	1
WH F104	gill	Nano	2	1
WH 25	gill	Nano	2	1
WHF93	gill	Nano	2	1
WH32	gill	Nano	2	1
WH105	gill	Nano	2	1
WH 429	gill	Nano	2	1
WH 20	gill	Nano	2	1
WH 26	kid	Nano	2	1
WH21	kid	Nano	2	0
WH 27	kid	Nano	2	1
WH 24	kid	Nano	2	1
WH 22	kid	Nano	2	1
WH 440	kid	Nano	2	0
WH 18	kid	Nano	2	0
WH 19	kid	Nano	2	0
WH 438	kid	Nano	2	0
WH 29	kid	Nano	2	1
WH 23	kid	Nano	2	1
WH 28	kid	Nano	2	1
WH 425	kid	Nano	2	1
WH 31	kid	Nano	2	1
WH 390	kid	Nano	2	0
WH 381	kid	Nano	2	1
WH 100	kid	Nano	2	1
WH 107	kid	Nano	2	1

id	organ	parasite	rep	pres
WH 96F	kid	Nano	2	1
WH F104	kid	Nano	2	1
WH 25	kid	Nano	2	1
WHF93	kid	Nano	2	1
WH32	kid	Nano	2	1
WH105	kid	Nano	2	1
WH 429	kid	Nano	2	1
WH 20	kid	Nano	2	0
WH 26	hrt	Nano	2	1
WH21	hrt	Nano	2	1
WH 24	hrt	Nano	2	0
WH 22	hrt	Nano	2	1
WH 18	hrt	Nano	2	1
WH 31	hrt	Nano	2	1
WH 390	hrt	Nano	2	1
WH 381	hrt	Nano	2	1
WH 100	hrt	Nano	2	1
WH 107	hrt	Nano	2	1
WH 96F	hrt	Nano	2	1
WH F104	hrt	Nano	2	1
WH 25	hrt	Nano	2	1
WHF93	hrt	Nano	2	1
WH32	hrt	Nano	2	0
WH 20	hrt	Nano	2	0
WH 26	kid	Furn	2	0
WH21	kid	Furn	2	0
WH 27	kid	Furn	2	0
WH 24	kid	Furn	2	0
WH 22	kid	Furn	2	0
WH 440	kid	Furn	2	0
WH 18	kid	Furn	2	0
WH 19	kid	Furn	2	0
WH 438	kid	Furn	2	0
WH 29	kid	Furn	2	0
WH 23	kid	Furn	2	0
WH 28	kid	Furn	2	0
WH 425	kid	Furn	2	0
WH 31	kid	Furn	2	0
WH 390	kid	Furn	2	0
WH 381	kid	Furn	2	0
WH 100	kid	Furn	2	0
WH 107	kid	Furn	2	0
WH 96F	kid	Furn	2	0

id	organ	parasite	rep	pres
WH F104	kid	Furn	2	0
WH 25	kid	Furn	2	0
WHF93	kid	Furn	2	0
WH32	kid	Furn	2	0
WH105	kid	Furn	2	0
WH 429	kid	Furn	2	0
WH 20	kid	Furn	2	0
WH 26	hrt	Furn	2	0
WH21	hrt	Furn	2	0
WH 27	hrt	Furn	2	0
WH 24	hrt	Furn	2	0
WH 22	hrt	Furn	2	0
WH 440	hrt	Furn	2	0
WH 18	hrt	Furn	2	0
WH 19	hrt	Furn	2	0
WH 438	hrt	Furn	2	0
WH 29	hrt	Furn	2	0
WH 23	hrt	Furn	2	0
WH 28	hrt	Furn	2	0
WH 425	hrt	Furn	2	0
WH 31	hrt	Furn	2	0
WH 390	hrt	Furn	2	0
WH 381	hrt	Furn	2	0
WH 100	hrt	Furn	2	0
WH 107	hrt	Furn	2	0
WH 96F	hrt	Furn	2	0
WH F104	hrt	Furn	2	0
WH 25	hrt	Furn	2	0
WHF93	hrt	Furn	2	0
WH32	hrt	Furn	2	0
WH105	hrt	Furn	2	0
WH 429	hrt	Furn	2	0
WH 20	hrt	Furn	2	0
WH 26	spl	Furn	2	0
WH21	spl	Furn	2	0
WH 27	spl	Furn	2	0
WH 24	spl	Furn	2	0
WH 22	spl	Furn	2	0
WH 440	spl	Furn	2	0
WH 18	spl	Furn	2	0
WH 19	spl	Furn	2	0
WH 438	spl	Furn	2	0
WH 29	spl	Furn	2	0

id	organ	parasite	rep	pres
WH 23	spl	Furn	2	0
WH 28	spl	Furn	2	0
WH 425	spl	Furn	2	0
WH 31	spl	Furn	2	0
WH 390	spl	Furn	2	0
WH 381	spl	Furn	2	0
WH 100	spl	Furn	2	0
WH 107	spl	Furn	2	0
WH 96F	spl	Furn	2	0
WH F104	spl	Furn	2	0
WH 25	spl	Furn	2	0
WHF93	spl	Furn	2	0
WH32	spl	Furn	2	0
WH105	spl	Furn	2	0
WH 429	spl	Furn	2	0
WH 20	spl	Furn	2	0
WH 26	liv	Furn	2	0
WH21	liv	Furn	2	0
WH 27	liv	Furn	2	0
WH 24	liv	Furn	2	0
WH 22	liv	Furn	2	0
WH 440	liv	Furn	2	0
WH 18	liv	Furn	2	0
WH 19	liv	Furn	2	0
WH 438	liv	Furn	2	0
WH 29	liv	Furn	2	0
WH 23	liv	Furn	2	0
WH 28	liv	Furn	2	0
WH 425	liv	Furn	2	0
WH 31	liv	Furn	2	0
WH 390	liv	Furn	2	0
WH 381	liv	Furn	2	0
WH 100	liv	Furn	2	0
WH 107	liv	Furn	2	0
WH 96F	liv	Furn	2	0
WH F104	liv	Furn	2	0
WH 25	liv	Furn	2	0
WHF93	liv	Furn	2	0
WH32	liv	Furn	2	0
WH105	liv	Furn	2	0
WH 429	liv	Furn	2	0
WH 20	liv	Furn	2	0
WH 26	gill	Furn	2	0

id	organ	parasite	rep	pres
WH21	gill	Furn	2	0
WH 27	gill	Furn	2	0
WH 24	gill	Furn	2	0
WH 22	gill	Furn	2	0
WH 440	gill	Furn	2	0
WH 18	gill	Furn	2	0
WH 19	gill	Furn	2	0
WH 438	gill	Furn	2	0
WH 29	gill	Furn	2	0
WH 23	gill	Furn	2	0
WH 28	gill	Furn	2	0
WH 425	gill	Furn	2	0
WH 31	gill	Furn	2	0
WH 390	gill	Furn	2	0
WH 381	gill	Furn	2	0
WH 100	gill	Furn	2	0
WH 107	gill	Furn	2	0
WH 96F	gill	Furn	2	0
WH F104	gill	Furn	2	0
WH 25	gill	Furn	2	0
WHF93	gill	Furn	2	0
WH32	gill	Furn	2	0
WH105	gill	Furn	2	0
WH 429	gill	Furn	2	0
WH 20	gill	Furn	2	0
WH 26	kid_glo	Pm	3	1
WH21	kid_glo	Pm	3	1
WH 27	kid_glo	Pm	3	1
WH 24	kid_glo	Pm	3	1
WH 22	kid_glo	Pm	3	0
WH 440	kid_glo	Pm	3	1
WH 18	kid_glo	Pm	3	1
WH 19	kid_glo	Pm	3	1
WH 438	kid_glo	Pm	3	1
WH 29	kid_glo	Pm	3	1
WH 23	kid_glo	Pm	3	1
WH 28	kid_glo	Pm	3	1
WH 425	kid_glo	Pm	3	1
WH 31	kid_glo	Pm	3	1
WH 390	kid_glo	Pm	3	0
WH 381	kid_glo	Pm	3	1
WH 100	kid_glo	Pm	3	1
WH 107	kid_glo	Pm	3	0

id	organ	parasite	rep	pres
WH 96F	kid_glo	Pm	3	1
WH F104	kid_glo	Pm	3	1
WH 25	kid_glo	Pm	3	0
WHF93	kid_glo	Pm	3	0
WH32	kid_glo	Pm	3	1
WH105	kid_glo	Pm	3	1
WH 20	kid_glo	Pm	3	1
WH 26	kid_tub	Pm	3	1
WH21	kid_tub	Pm	3	0
WH 27	kid_tub	Pm	3	1
WH 24	kid_tub	Pm	3	1
WH 22	kid_tub	Pm	3	1
WH 440	kid_tub	Pm	3	0
WH 18	kid_tub	Pm	3	1
WH 19	kid_tub	Pm	3	1
WH 438	kid_tub	Pm	3	1
WH 29	kid_tub	Pm	3	1
WH 23	kid_tub	Pm	3	1
WH 28	kid_tub	Pm	3	1
WH 425	kid_tub	Pm	3	1
WH 31	kid_tub	Pm	3	0
WH 390	kid_tub	Pm	3	1
WH 381	kid_tub	Pm	3	1
WH 100	kid_tub	Pm	3	1
WH 107	kid_tub	Pm	3	1
WH 96F	kid_tub	Pm	3	1
WH F104	kid_tub	Pm	3	1
WH 25	kid_tub	Pm	3	0
WHF93	kid_tub	Pm	3	1
WH32	kid_tub	Pm	3	1
WH105	kid_tub	Pm	3	0
WH 20	kid_tub	Pm	3	1
WH 26	gill	AE	3	0
WH21	gill	AE	3	0
WH 27	gill	AE	3	1
WH 24	gill	AE	3	0
WH 22	gill	AE	3	0
WH 440	gill	AE	3	0
WH 18	gill	AE	3	0
WH 19	gill	AE	3	0
WH 438	gill	AE	3	1
WH 29	gill	AE	3	1
WH 23	gill	AE	3	0

id	organ	parasite	rep	pres
WH 28	gill	AE	3	0
WH 425	gill	AE	3	1
WH 31	gill	AE	3	0
WH 390	gill	AE	3	0
WH 381	gill	AE	3	0
WH 100	gill	AE	3	0
WH 107	gill	AE	3	0
WH 96F	gill	AE	3	0
WH F104	gill	AE	3	0
WH 25	gill	AE	3	1
WHF93	gill	AE	3	0
WH32	gill	AE	3	0
WH105	gill	AE	3	1
WH 429	gill	AE	3	0
WH 20	gill	AE	3	1
WH 26	kid	RS	3	0
WH21	kid	RS	3	0
WH 27	kid	RS	3	0
WH 24	kid	RS	3	0
WH 22	kid	RS	3	0
WH 440	kid	RS	3	0
WH 18	kid	RS	3	0
WH 19	kid	RS	3	0
WH 438	kid	RS	3	0
WH 29	kid	RS	3	0
WH 23	kid	RS	3	0
WH 28	kid	RS	3	0
WH 425	kid	RS	3	0
WH 31	kid	RS	3	0
WH 390	kid	RS	3	0
WH 381	kid	RS	3	0
WH 100	kid	RS	3	0
WH 107	kid	RS	3	0
WH 96F	kid	RS	3	0
WH F104	kid	RS	3	0
WH 25	kid	RS	3	0
WHF93	kid	RS	3	0
WH32	kid	RS	3	1
WH105	kid	RS	3	1
WH 429	kid	RS	3	0
WH 20	kid	RS	3	0
WH 26	liv	RS	3	0
WH21	liv	RS	3	0

id	organ	parasite	rep	pres
WH 27	liv	RS	3	0
WH 24	liv	RS	3	0
WH 22	liv	RS	3	0
WH 440	liv	RS	3	0
WH 18	liv	RS	3	0
WH 19	liv	RS	3	0
WH 438	liv	RS	3	0
WH 29	liv	RS	3	0
WH 23	liv	RS	3	0
WH 28	liv	RS	3	0
WH 425	liv	RS	3	0
WH 31	liv	RS	3	0
WH 390	liv	RS	3	0
WH 381	liv	RS	3	0
WH 100	liv	RS	3	0
WH 107	liv	RS	3	0
WH 96F	liv	RS	3	0
WH F104	liv	RS	3	0
WH 25	liv	RS	3	0
WHF93	liv	RS	3	0
WH32	liv	RS	3	0
WH105	liv	RS	3	0
WH 20	liv	RS	3	0
WH 26	spl	RS	3	0
WH21	spl	RS	3	0
WH 27	spl	RS	3	0
WH 24	spl	RS	3	0
WH 22	spl	RS	3	0
WH 440	spl	RS	3	0
WH 18	spl	RS	3	0
WH 19	spl	RS	3	0
WH 438	spl	RS	3	0
WH 29	spl	RS	3	0
WH 23	spl	RS	3	0
WH 28	spl	RS	3	0
WH 425	spl	RS	3	0
WH 31	spl	RS	3	0
WH 390	spl	RS	3	0
WH 381	spl	RS	3	0
WH 100	spl	RS	3	0
WH 107	spl	RS	3	0
WH 96F	spl	RS	3	0
WH F104	spl	RS	3	0

id	organ	parasite	rep	pres
WH 25	spl	RS	3	0
WHF93	spl	RS	3	0
WH32	spl	RS	3	0
WH105	spl	RS	3	0
WH 20	spl	RS	3	0
WH 26	gill	Nano	3	1
WH21	gill	Nano	3	0
WH 27	gill	Nano	3	1
WH 24	gill	Nano	3	1
WH 22	gill	Nano	3	1
WH 440	gill	Nano	3	1
WH 18	gill	Nano	3	1
WH 19	gill	Nano	3	1
WH 438	gill	Nano	3	1
WH 29	gill	Nano	3	1
WH 23	gill	Nano	3	0
WH 28	gill	Nano	3	1
WH 425	gill	Nano	3	1
WH 31	gill	Nano	3	0
WH 390	gill	Nano	3	1
WH 381	gill	Nano	3	1
WH 100	gill	Nano	3	1
WH 107	gill	Nano	3	1
WH 96F	gill	Nano	3	1
WH F104	gill	Nano	3	1
WH 25	gill	Nano	3	1
WHF93	gill	Nano	3	1
WH32	gill	Nano	3	1
WH105	gill	Nano	3	1
WH 429	gill	Nano	3	0
WH 20	gill	Nano	3	1
WH 26	kid	Nano	3	1
WH21	kid	Nano	3	1
WH 27	kid	Nano	3	1
WH 24	kid	Nano	3	1
WH 22	kid	Nano	3	1
WH 440	kid	Nano	3	1
WH 18	kid	Nano	3	1
WH 19	kid	Nano	3	1
WH 438	kid	Nano	3	1
WH 29	kid	Nano	3	0
WH 23	kid	Nano	3	0
WH 28	kid	Nano	3	1

id	organ	parasite	rep	pres
WH 425	kid	Nano	3	1
WH 31	kid	Nano	3	1
WH 390	kid	Nano	3	1
WH 381	kid	Nano	3	1
WH 100	kid	Nano	3	1
WH 107	kid	Nano	3	1
WH 96F	kid	Nano	3	1
WH F104	kid	Nano	3	0
WH 25	kid	Nano	3	0
WHF93	kid	Nano	3	1
WH32	kid	Nano	3	1
WH105	kid	Nano	3	1
WH 20	kid	Nano	3	0
WH 26	hrt	Nano	3	1
WH21	hrt	Nano	3	1
WH 24	hrt	Nano	3	0
WH 22	hrt	Nano	3	1
WH 31	hrt	Nano	3	1
WH 390	hrt	Nano	3	1
WH 381	hrt	Nano	3	1
WH 100	hrt	Nano	3	1
WH 107	hrt	Nano	3	1
WH 96F	hrt	Nano	3	1
WH F104	hrt	Nano	3	1
WH 25	hrt	Nano	3	1
WHF93	hrt	Nano	3	1
WH32	hrt	Nano	3	1
WH105	hrt	Nano	3	0
WH 20	hrt	Nano	3	0
WH 26	kid	Furn	3	0
WH21	kid	Furn	3	0
WH 27	kid	Furn	3	0
WH 24	kid	Furn	3	0
WH 22	kid	Furn	3	0
WH 440	kid	Furn	3	0
WH 18	kid	Furn	3	0
WH 19	kid	Furn	3	0
WH 438	kid	Furn	3	0
WH 29	kid	Furn	3	0
WH 23	kid	Furn	3	0
WH 28	kid	Furn	3	0
WH 425	kid	Furn	3	0
WH 31	kid	Furn	3	0

id	organ	parasite	rep	pres
WH 390	kid	Furn	3	0
WH 381	kid	Furn	3	0
WH 100	kid	Furn	3	0
WH 107	kid	Furn	3	0
WH 96F	kid	Furn	3	0
WH F104	kid	Furn	3	0
WH 25	kid	Furn	3	0
WHF93	kid	Furn	3	0
WH32	kid	Furn	3	0
WH105	kid	Furn	3	0
WH 429	kid	Furn	3	0
WH 20	kid	Furn	3	0
WH 26	hrt	Furn	3	0
WH21	hrt	Furn	3	0
WH 27	hrt	Furn	3	0
WH 24	hrt	Furn	3	0
WH 22	hrt	Furn	3	0
WH 440	hrt	Furn	3	0
WH 18	hrt	Furn	3	0
WH 19	hrt	Furn	3	0
WH 438	hrt	Furn	3	0
WH 29	hrt	Furn	3	0
WH 23	hrt	Furn	3	0
WH 28	hrt	Furn	3	0
WH 425	hrt	Furn	3	0
WH 31	hrt	Furn	3	0
WH 390	hrt	Furn	3	0
WH 381	hrt	Furn	3	0
WH 100	hrt	Furn	3	0
WH 107	hrt	Furn	3	0
WH 96F	hrt	Furn	3	0
WH F104	hrt	Furn	3	0
WH 25	hrt	Furn	3	0
WHF93	hrt	Furn	3	0
WH32	hrt	Furn	3	0
WH105	hrt	Furn	3	0
WH 429	hrt	Furn	3	0
WH 20	hrt	Furn	3	0
WH 26	spl	Furn	3	0
WH21	spl	Furn	3	0
WH 27	spl	Furn	3	0
WH 24	spl	Furn	3	0
WH 22	spl	Furn	3	0

id	organ	parasite	rep	pres
WH 440	spl	Furn	3	0
WH 18	spl	Furn	3	0
WH 19	spl	Furn	3	0
WH 438	spl	Furn	3	0
WH 29	spl	Furn	3	0
WH 23	spl	Furn	3	0
WH 28	spl	Furn	3	0
WH 425	spl	Furn	3	0
WH 31	spl	Furn	3	0
WH 390	spl	Furn	3	0
WH 381	spl	Furn	3	0
WH 100	spl	Furn	3	0
WH 107	spl	Furn	3	0
WH 96F	spl	Furn	3	0
WH F104	spl	Furn	3	0
WH 25	spl	Furn	3	0
WHF93	spl	Furn	3	0
WH32	spl	Furn	3	0
WH105	spl	Furn	3	0
WH 429	spl	Furn	3	0
WH 20	spl	Furn	3	0
WH 26	liv	Furn	3	0
WH21	liv	Furn	3	0
WH 27	liv	Furn	3	0
WH 24	liv	Furn	3	0
WH 22	liv	Furn	3	0
WH 440	liv	Furn	3	0
WH 18	liv	Furn	3	0
WH 19	liv	Furn	3	0
WH 438	liv	Furn	3	0
WH 29	liv	Furn	3	0
WH 23	liv	Furn	3	0
WH 28	liv	Furn	3	0
WH 425	liv	Furn	3	0
WH 31	liv	Furn	3	0
WH 390	liv	Furn	3	0
WH 381	liv	Furn	3	0
WH 100	liv	Furn	3	0
WH 107	liv	Furn	3	0
WH 96F	liv	Furn	3	0
WH F104	liv	Furn	3	0
WH 25	liv	Furn	3	0
WHF93	liv	Furn	3	0

id	organ	parasite	rep	pres
WH32	liv	Furn	3	0
WH105	liv	Furn	3	0
WH 429	liv	Furn	3	0
WH 20	liv	Furn	3	0
WH 26	gill	Furn	3	0
WH21	gill	Furn	3	0
WH 27	gill	Furn	3	0
WH 24	gill	Furn	3	0
WH 22	gill	Furn	3	0
WH 440	gill	Furn	3	0
WH 18	gill	Furn	3	0
WH 19	gill	Furn	3	0
WH 438	gill	Furn	3	0
WH 29	gill	Furn	3	0
WH 23	gill	Furn	3	0
WH 28	gill	Furn	3	0
WH 425	gill	Furn	3	0
WH 31	gill	Furn	3	0
WH 390	gill	Furn	3	0
WH 381	gill	Furn	3	0
WH 100	gill	Furn	3	0
WH 107	gill	Furn	3	0
WH 96F	gill	Furn	3	0
WH F104	gill	Furn	3	0
WH 25	gill	Furn	3	0
WHF93	gill	Furn	3	0
WH32	gill	Furn	3	0
WH105	gill	Furn	3	0
WH 429	gill	Furn	3	0
WH 20	gill	Furn	3	0
